# Increased Growth and Germination Success in Plants following Hydrogen Sulfide Administration

**DOI:** 10.1371/journal.pone.0062048

**Published:** 2013-04-17

**Authors:** Frederick D. Dooley, Suven P. Nair, Peter D. Ward

**Affiliations:** Department of Biology, University of Washington, Seattle, Washington, United States of America; Fred Hutchinson Cancer Research Center, United States of America

## Abstract

This study presents a novel way of enhancing plant growth through the use of a non-petroleum based product. We report here that exposing either roots or seeds of multicellular plants to extremely low concentrations of dissolved hydrogen sulfide at any stage of life causes statistically significant increases in biomass including higher fruit yield. Individual cells in treated plants were smaller (∼13%) than those of controls. Germination success and seedling size increased in, bean, corn, wheat, and pea seeds while time to germination decreases. These findings indicated an important role of H_2_S as a signaling molecule that can increase the growth rate of all species yet tested. The increased crop yields reported here has the potential to effect the world's agricultural output.

## Introduction

The biological effects of hydrogen sulfide (H_2_S) have received increasing attention in recent years, not only as a putative kill mechanism during past mass extinctions [1]–[4], but also as an important signaling molecule in both aerobic and anaerobic organisms [5]–[13]. Hydrogen sulfide has recently been added to nitric oxide (NO) and carbon monoxide (CO) as a newly categorized group of biologically active gases termed gasotransmitters and gasomediators [7], [14]. The origin of these dual activities remains unknown, but it may be that these varied signaling and biological mediating capabilities are remnants of biological responses by life either evolving or inhabiting highly sulfidic and anoxic environments of earlier times in Earth history. Today, H_2_S causes a wide variety of vital effects across the “Tree of Life”, from metabolic inhibition [15], to energy source [16]–[20], to coordination of developmental growth programs in yeast [21]–[22] and perhaps higher organisms as well. These findings led Lloyd [16] to propose that H_2_S is as an important signaling mediator in most or all prokaryotic clades. Yet, in spite of these findings, only recently have there been analogous investigations into the potential signaling role of H_2_S in eukaryotes. To date, only studies on its effect on the functioning of the electron transport chain in animals, and separately on its inhibition of physiological processes enabling endothermy in birds and mammals have been conducted [23]–[24].

Studies into the effects of sulfur and sulfide compounds on plants are still few in number [13], [25]–[26], and of these, most have concerned the lethal effects of gaseous hydrogen sulfide on plants [13], [26]. From these it is now known that H_2_S causes inhibition of photosynthesis at high concentrations [25], [27]–[29] and that it can decrease the time to germination [30], but also increases the resilience to drought and heavy metal toxicity [30]–[33]. Recent emerging evidence has also suggested a possible signaling role for stomatal apertures [11]–[12], [26], and in promoting chloroplast biogenesis [25].

To ascertain the potential role of H_2_S in that of higher plants, we conducted a series of experiments designed to evaluate potential effects of H_2_S on various plant species when administered at sub-lethal levels. We show here that micromolar concentrations of hydrogen sulfide dissolved in water and taken up by either seeds or roots have significant effects on important aspects of plant physiology and life history, and may be an important new way of increasing human crop yields.

## Results

### Hydroponic Seed and Seedling Trials

**Figure 1:**
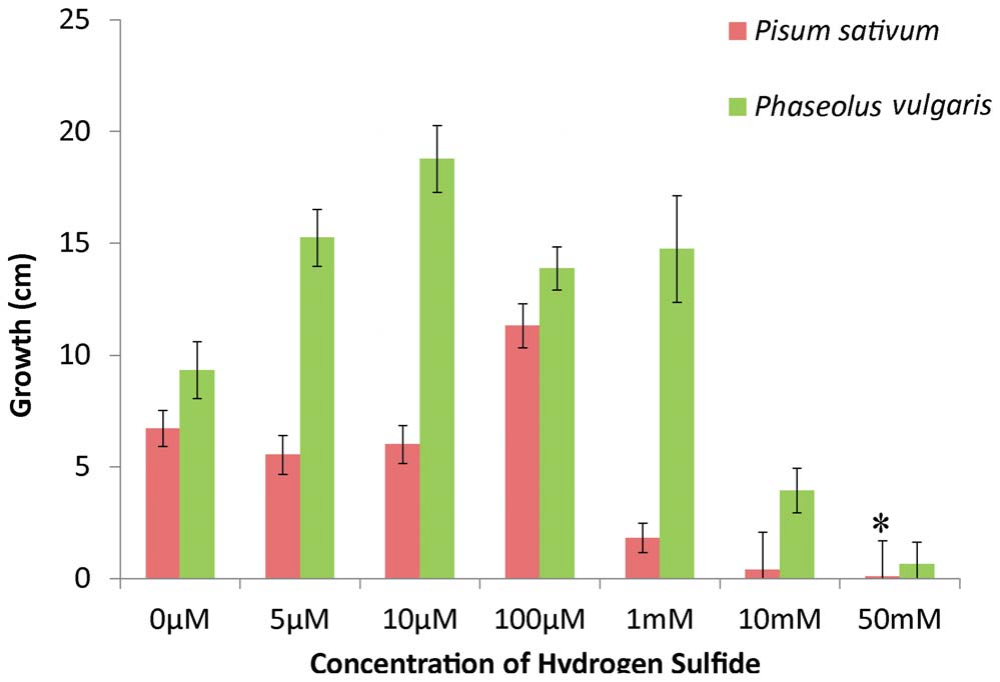
In both *Pisum sativus* (pea plants) *and Phaseolus vulgaris* (bean plants), increased growth as measured linear growth (stems plus roots) is observed with all levels of H_2_S exposures. * signifies a data point as being ≤0. SD bars shown.

Both root systems and seeds of *Phaseolus vulgaris* (*Bean*), *Pisum sativum* (Pea), *USU-Apogee* (Space Wheat), and *Zea mays L*. (Corn) were exposed to variable concentrations of H_2_S dissolved in deionized water. Concentrations of H_2_S varying from 10–100 µM caused absolute stem and leaf growth (both length and mass) in seedlings to be significantly larger (F = 10.86, df 96, P<0.001) for all treated plants than controls of the same species (Figure 1). Maximum growth rates for beans was highest at 10 µM, with an average length of 18.78±1.49 cm, which was significantly higher than controls at 8.8±1.26 cm (T = 3.8062, df 49, P<0.001). Additionally, maximum wet mass, with an average of 0.951±0.16 g (T = 5.46, P<0.001; F = 9.58, df 96, P<0.001), was observed in 10 µM treatment. The 5 µM treatment was similar to the 10 µM treatment at 15.24±1.27 cm and 0.928±0.10 g, but was still significantly larger than the controls (F = 10.49, df 96, P<0.001). For peas, maximum length change (11.3±0.66 cm) occurred at 100 µM (Figure 1) and was the only treatment that was statistically different (T = 3.4035 P<0.01 & F = 3.59, df 1, P<0.01) from the controls (6.72±0.8 cm). However, maximum change in wet mass occurred at 5 µM with an observed value 0.223±0.04 g, which was statistically different from controls 0.04±0.05 g (F = 4.494, df 96, P<0.01). Plants treated with higher than 1 mM experienced decreased growth rate, with mortality at >20 mM.

**Figure 2:**
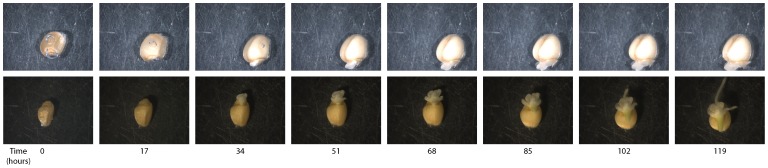
This image shows the germination of a *USU-Apogee* (Space Wheat) seed taken over 119 hrs at 16 hr intervals. The top panel shows the control seed germination while the bottom displays the H_2_S exposed seed. Note at 119 hrs the control is less developed then the exposed.

In addition to increasing growth rates at a finite and relatively narrow concentration of H_2_S, we found that time to germination in seeds treated with H_2_S was significantly less than values observed in untreated seeds (controls) (Figure 2). Seedlings of treated seeds also showed a significantly greater length compared to controls after seven days (Table 1).

**Table 1:** Percent Germination over time by concentration of H_2_S.

Zea mays L. (corn)					
Time (Hours)	0( µM)	10( µM)	100( µM)	500( µM)	1(mM)
24	0	0	0	0	0
48	3.3	10	26.6	5	0
72	23.3	40	46.6	45	40
168	90	90	93	60	75
USU-apogee (wheat)					
24	80	95	90	55	60
48	90	100	95	85	65
72	95	100	95	95	100
168	95	100	95	100	100
Pisum sativum (pea)					
24	0	6.6	10	5	5
48	50	73.3	63.3	30	25
72	73.3	90	76.6	85	65
168	80	96.6	90	85	65
Phaseolus vulgaris (bean)					
24	3.3	6.6	10	10	5
48	3.3	10	13	15	15
72	16.6	26.6	30	75	55
168	93.3	90	100	100	95

### Growth To Maturity In Soil

**Figure 3:**
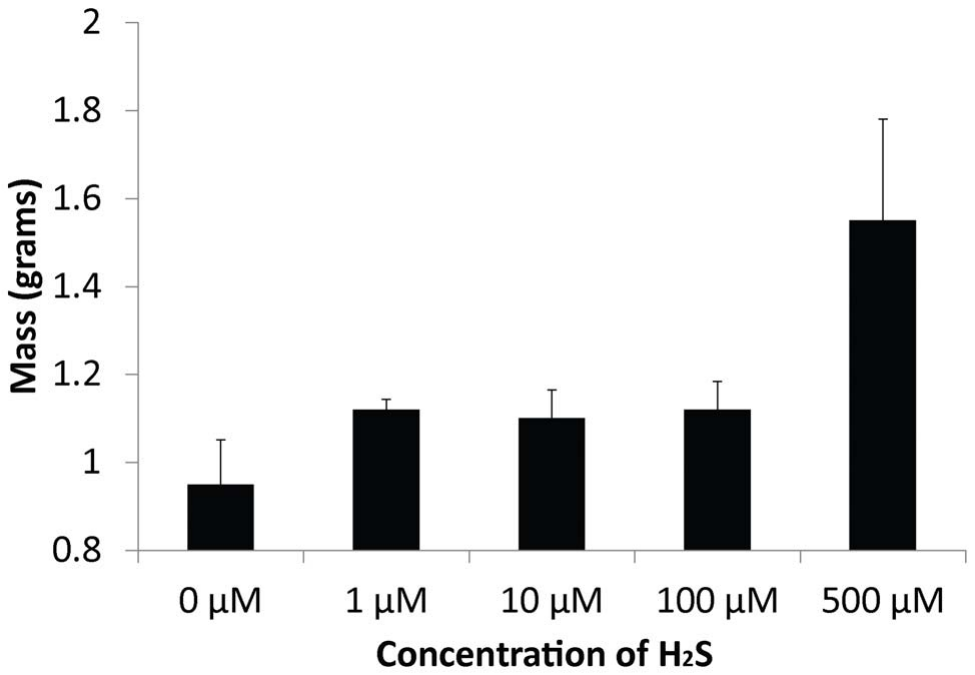
Average fruit yield per wheat plant. SD bars shown.

When space wheat plants were grown to maturity, it was observed that the overall length of the plants, when exposed to H_2_S, were slightly longer, however not statistically different than that of the controls (37.1±2.2 cm for 0 µM (controls), whereas 1 µM was 37.5±4.1 cm, 10 µM was 38.4±3.6 cm, 100 µM was 37.2±4.6 cm, and 500 µM was 37.2±4.7 cm). This difference between treatments is amplified when exposing the plants to H_2_S every seven days (36.6±2.1 cm for 0 µM (controls), whereas 1 µM was 38.6±5.2 cm, 10 µM was 39.9±2.1 cm, 100 µM was 40.3±3.6 cm, and 500 µM was 40.2±1.8 cm; P = 0.08; F = 2.6). The overall mass of the plant, roots and fruit were independently larger than the controls for all plants exposed to H_2_S. The mass of the entire plant was 7.0±0.3 g for 0 µM (controls), whereas 1 µM was 8.3±0.6 g, 10 µM was 9.9±0.5 g, 100 µM was 8.8±0.7 g, and 500 µM was 10.8±0.8 g, and each was statistically different from the controls (df = 84, F = 4.81; P<0.01). Likewise, the mass of the roots was larger in treated plants (1 µM was 3.8±1.6 g, 10 µM was 4.5±0.8 g, 100 µM was 4.7±1.0 g, and 500 µM was 4.7±0.4 g) compared to that of the controls (2.8±0.6 g for 0 µM). Beyond the macro plant, the produced fruit was larger in the treated plants: 0.95±0.1 g for 0 µM (controls), whereas 1 µM was 1.1±0.0 g, 10 µM was 1.1±0.1 g, 100 µM was 1.1±0.1 g, and 500 µM was 1.5±0.2 g (Figure 3).

### Leaf Disk Trials

Leaf disks exposed to H_2_S also experienced increased growth relative to controls. (F = 5.16, df 63, P<0.01). Mean change in growth was 0.17±0.02 mm for controls; 1 µM: 0.36±0.01 mm; 10 µM: 0.26±.03 mm; and 100 µM: 0.32±0.01 mm. Finally, when we compared the cells from the leaf disks it was determined that the cell size (diameter and area) was significantly smaller (∼13% and 18%) than those of the controls (F = 66.7, df 1590, P<0.001). Mean diameter of controls was 8.38±0.07 µm; 1 µM: 7.4±0.09 µm; 10 µM: 7.1±0.06 µm; and 100 µM: 7.3±0.05 µm.

### Photosynthetic Activity

Throughout the experiments, photosynthetic activity (Qmax - a measurement of maximum photosynthetic output) of all plants was monitored using a Fluorcam. Qmax values maintained relatively comparable levels (0.6–0.7 for all treatments) to untreated controls until the 50 mM concentration, where significant decrease (<0.1) was observed and differences in reflected spectra were apparent [*e.g*. 29]. Additionally, high (<10 mM) but sub-lethal levels caused photosystem (PS) II to shut down, whereas PS I remained active through the experimental period through and up to plant senescence [29].

## Discussion

In recent years, numerous experiments have shown that hydrogen sulfide causes an array of biological effects [13], [33]–[38] and in this study we add to that growing list. Our experiments show that the application of extremely narrow concentrations (at taxon specific levels) of liquid H_2_S produces two separate kinds of increased growth rates in plants: time to seed germination; absolute mass of tissue in roots, stems, and leaves. Unlike the results in a previous study [33], our applications had no adverse effects on any of the treated plants: for example we observed no lesions or tumors during the duration of the studies reported here. Enhanced growth continued for up to seven days after a single exposure to H_2_S, followed by a return to the slower growth rates observed in controls, unless re-exposed. This study also shows that H_2_S is the *only* chemical necessary to produce such large differences in growth rates in these species.

Regardless of the physical, “macro-” changes in the plant, it is clear that the causes of these results are occurring on a cellular level. Our observation that cells increase in number rather than size suggests that the H_2_S molecules are provoking cellular division through some signaling process. The increased photosynthetic activity shown in a previous study [29] is either caused by increasing the photo-efficiency in the existing chloroplasts, or by increasing the absolute number of chloroplasts per area [25].

Because hydrogen sulfide has ‘clandestic messenger’ like properties [13], this combination of multiple cellular effects (changes in photosynthetic activity and cellular growth rate) from the plant's contact with the relatively few number of H_2_S molecules affecting the plants treated in this study is reasonable. We hypothesize that administered H_2_S could be regulating a hormonal pathway [11], [26] or actively effecting a transcription factor involved in cellular replication [39], and not just increasing growth rate as a byproduct of the addition of sulfur as a nutrient (“fertilizer”), as is seen through addition of large concentrations of phosphates or nitrates. In any event, it is clear that H_2_S is affecting the basic biology of the plants.

The origin of these effects is open to speculation. However, it may be that an increase in growth rates of some plants at the early onset of an increasing atmospheric or aqueous H_2_S load was an evolutionary response to short-term and catastrophic changes in global atmosphere during Phanerozoic mass extinction events, when a combination of higher temperature, oceanic anoxia, and release of gaseous hydrogen sulfide into the atmosphere contributed to various global mass extinctions [1]–[3]. Previous work on H_2_S toxicity (*e.g*. [29], and unpublished observations made during the research described here) has shown that toxicity (as recognized by LD_50_ curves) decreases with plant size, even in the same species. The mechanism observed here might be that simple: at the first recognition of oncoming H_2_S concentrations, survivability would be affected by overall plant size. Rapid growth would be selected for. While these results are early in development, these findings may provide beneficial use to agriculture and biofuels.

## Materials and Methods

### Hydrogen Sulfide Preparation

Half-molar hydrogen sulfide was made by dissolving 78.04 grams of anhydrous sodium sulfide into 500 mL of double filtered di-water. Hydrochloric acid was then titrated into this solution in 0.01 mL increments while stirring until pH of 7.2 was reached, resulting in a solution 0.5 M±25 mM (5%) as determined by using H_2_S/Sulfide Probe (Sea & Sun Technology GmbH, Trappenkamp, Germany). The 0.5 M H_2_S solution was then filtered and stored in a 500 mL flask filled with nitrogen gas to maintain stability.

Each treatment concentration was made adding ddi-H_2_O. After dilution the H_2_S/Sulfide Probe was used to confirm concentration.

### Hydroponic Seed and Seedling Trials

Seeds from four common crop species *Phaseolus vulgaris* (Bean), *Pisum sativum* (Pea), *USU-Apogee* (Space Wheat), and *Zea mays L*. (Corn) were exposed to five levels (0 µM, 10 µM, 100 µM, 500 µM and 1 mM,±5 µM error) of liquid hydrogen sulfide dissolved in ddi-H_2_O. Germination rates as well as the quantity of growth was measured and compared to a control (0 µM H_2_S), for a seven day growth period. Thirty seeds, of each species were placed into 100 ml petri-dishes filled with 50 ml of solution. Due to the relatively short half-life of H_2_S when in solution [40], treatment liquids were replaced daily. Petri-dishes containing seeds were randomly placed on a shelf serviced by 12 hours of ∼100 µmol m^−2^s^−1^ of light daily. Temperature was ambient room temperature (∼23°C). Every day at the same hour, the number of seeds which germinated was recorded. At 72 and 168 hours, the length of the fresh seedling was recorded using digital calipers

Seedlings of the same species of pea and bean used in the germination trials were also germinated hydroponically in 250 mL containers (seeds in this case untreated with H_2_S) Seeds were wrapped with a clean paper-towel and mounted to a glass slide. The slide, containing a seed, was placed into the container and immersed in 75 ml of ddi-H_2_O. The containers were randomly placed on shelves serviced by grow lights which provided 12 hours of ∼100 µmol m^−2^s^−1^ of light daily. Water was replenished weekly. Temperature was ambient room temperature (∼23°C).

Upon germination, seedlings were grown for fourteen days and then randomly selected for treatments. Twenty-Five seedlings were selected for each treatment and a control (0 µM, 5 µM, 10 µM, 100 µM, 1 mM, 10 mM and 50 mM). Before exposure, and 24, 48 & 168 hours, photosynthetic output (Qmax of the formula Fv/Fm) was measured with a Z100 Kinetic Multispectral Fluorescence Imaging FluorCam System by P.S.I. Qmax, the maximal photochemical efficiency of PSII (Fν/Fm) was calculated according to Krause and Weis [41] equation:
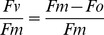
. At the same time, length measurements of both the shoot and root, and wet mass of each plant were taken and recorded. A liquid H_2_S solution of 5 µM, 10 µM, 100 µM, 1 mM, 10 mM, and 50 mM was then applied to the root each day. All plants were raised under similar environmental conditions ∼100 µmol m^−2^s^−1^ of light daily, ambient room temperature (∼23°C) and relative humidity. In addition, we measured the pH of each treatment in order to rule out pH change as the reasoning for growth differences.

### Growth To Maturity In Soil

One seed, of *USU-Apogee*, was placed in each of the four corners of a half-gallon pot filled with 400 grams of sunshine #6 soil. Each seed was submersed one cm from the surface of the soil. Six pots, each containing four seeds, were placed into each treatment. Treatments consisted of 0, 1, 10, 100 and 500 µM H_2_S solutions (diluted with ddi-H_2_O). Additionally, each treatment consisted of two sub treatments; a three day and weekly exposure regimen.

Seeded pots were watered, either weekly or every three days, with 300 milliliters of the corresponding treatment solutions (0, 1, 10, 100 and 500 µM). Seeded pots were haphazardly distributed in the University of Washington Botany Greenhouse. Each week the length of the shoot, number of leaves, and developmental level was measured. At the conclusion of nine weeks all plants were mature and had produced fruit. Each plant was carefully removed from the soil with root mass intact, and gently washed to remove the dirt. Whole plants were patted dry with a paper towel and weighed to the nearest 0.1grams. The roots and the fruit were independently removed from the plant and weighed to the nearest 0.1grams. Finally, the length of the plant was measured (to the nearest 0.5 cm).

### Leaf Disks

Sixty-four, 6.05±0.03 millimeter diameter, leaf disks were cut from growing leaves on a bean plant, which was grown in UW Botany Greenhouse. Disks were floated in a deionized water bath. Individual disks were haphazardly selected from this water bath, measured with digital calipers and put into labeled petri-dishes. Numbers associated with the labeled petri-dishes were randomly selected using a number generator and sixteen individual leaf disks were placed into treatments (0, 1, 10 and 100 µM of H_2_S + ddi-H_2_O soln.) by a second individual. The first person, who took measurements, was unaware of which disks were in what treatment throughout the experiment. Initial disk size per treatment was statistically compared using ANOVA, to determine if the sample was truly random. Twenty-four and forty-eight hours after exposure, leaf disks which were determined to be random were re-measured, using digital calipers, and the growth rate was compared.

Images (taken from Leica dissection scope camera) from an additional replicate of 10 disks per treatment, using the same double blind methods, were analyzed with Mitotic Image Plus 2.0 (Motic China Group Co.). Measurements consisted of: precision diameter (the largest distance across the leaf using two points); 3-point circle (3 points selected at random to form a circle of best fit); 5-point circle (5 points selected at random to form a circle of best fit); area in field of view using background exclusion. Measurements were compared to that of the calipers and differences were compared by using theoretical calculations: i.e. a theoretical area was calculated using the diameter (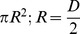)as determined by the calipers and compared to that of the measurements obtained above.

Individual cell size was measured using tissues obtained from the leaf disk experiments. Cell size was measured using a 40× lens on compound microscope with camera attached. Images obtained from the microscope were analyzed using Motic Images Plus 2.0. Diameter and area of all cells, in the field of view, were measured; a total of 1590 cells.

### Statistical Test

Means from each treatment group were compared against the control by a paired T-test. Secondly, these data was inputted into R (R version 2.14.2) and treatments were compared as factors using a linear regression model with ANOVA. Both T and F statistics are listed when appropriate.
